# Effectiveness of SBIRT for Alcohol Use Disorders in the Emergency Department: A Systematic Review

**DOI:** 10.5811/westjem.2017.7.34373

**Published:** 2017-09-21

**Authors:** Isabel A. Barata, Jamie R. Shandro, Margaret Montgomery, Robin Polansky, Carolyn J. Sachs, Herbert C. Duber, Lindsay M. Weaver, Alan Heins, Heather S. Owen, Elaine B. Josephson, Wendy Macias-Konstantopoulos

**Affiliations:** * North Shore University Hospital, Department of Emergency Medicine, Manhasset, New York; † Donald and Barbara Zucker School of Medicine at Hofstra/Northwell, Hempstead, New York; ‡ Harborview Medical Center, University of Washington Medical Center, Department of Emergency Medicine, Seattle, Washington; § American College of Emergency Physicians, Dallas, Texas; ¶ Cedars-Sinai Medical Center, Department of Emergency Medicine, Los Angeles, California; || UCLA David Geffen School of Medicine, Emergency Medicine Center, Los Angeles, California; # Indiana University School of Medicine, Department of Emergency Medicine, Indianapolis, Indiana; ** Cullman Regional Medical Center, Department of Emergency Medicine, Cullman, Alabama; †† Parkland Memorial Hospital, Department of Emergency Medicine, Dallas, Texas; ‡‡ Lincoln Medical and Mental Health Center, Department of Emergency Medicine, Bronx, New York; §§ Massachusetts General Hospital, Department of Emergency Medicine, Boston, Massachusetts

## Abstract

**Introduction:**

Alcohol use disorders (AUD) place a significant burden on individuals and society. The emergency department (ED) offers a unique opportunity to address AUD with brief screening tools and early intervention. We undertook a systematic review of the effectiveness of ED brief interventions for patients identified through screening who are at risk for AUD, and the effectiveness of these interventions at reducing alcohol intake and preventing alcohol-related injuries.

**Methods:**

We conducted systematic electronic database searches to include randomized controlled trials of AUD screening, brief intervention, referral, and treatment (SBIRT), from January 1966 to April 2016. Two authors graded and abstracted data from each included paper.

**Results:**

We found 35 articles that had direct relevance to the ED with enrolled patients ranging from 12 to 70 years of age. Multiple alcohol screening tools were used to identify patients at risk for AUD. Brief intervention (BI) and brief motivational intervention (BMI) strategies were compared to a control intervention or usual care. Thirteen studies enrolling a total of 5,261 participants reported significant differences between control and intervention groups in their main alcohol-outcome criteria of number of drink days and number of units per drink day. Sixteen studies showed a reduction of alcohol consumption in both the control and intervention groups; of those, seven studies did not identify a significant intervention effect for the main outcome criteria, but nine observed some significant differences between BI and control conditions for specific subgroups (i.e., adolescents and adolescents with prior history of drinking and driving; women 22 years old or younger; low or moderate drinkers); or secondary outcome criteria (e.g. reduction in driving while intoxicated).

**Conclusion:**

Moderate-quality evidence of targeted use of BI/BMI in the ED showed a small reduction in alcohol use in low or moderate drinkers, a reduction in the negative consequences of use (such as injury), and a decline in ED repeat visits for adults and children 12 years of age and older. BI delivered in the ED appears to have a short-term effect in reducing at-risk drinking.

## INTRODUCTION

The literature refers to harmful, hazardous, and risky drinking interchangeably as a pattern of drinking that increases risk of harm for the person consuming alcohol and/or others.^1^ Alcohol dependence is a result of repeated use leading to a person having impaired control over the use of alcohol despite physical, psychological, and social harms.^2^ The fifth edition of the *Diagnostic and Statistical Manual* (DSM–5) integrates alcohol abuse and alcohol dependence into a single disorder called alcohol use disorder (AUD), with mild, moderate, and severe sub-classifications.^3^

Excess alcohol consumption places a significant burden on individuals and society. The majority of adult patients in the United States consume alcohol with a 71% one-year and 57% one-month prevalence reported by those over age 18.^4^ Another 24.7% report binge drinking and 6.7% report heavy drinking.^5^ Moreover, 16.3 million adults, 6.8% of the U.S. population meet criteria for an AUD.^6^ Only 8.9% of the 16.3 million with AUD (i.e., about 1.5 million) received treatment for an AUD at a specialized facility.^7^ The 2012–2013 National Epidemiologic Survey on Alcohol and Related Conditions III (NESARC-III) found that the lifetime prevalence of AUD was 29.1%, with only 19.8% of respondents with lifetime AUD having ever been treated.^8^ In 2014, an estimated 679,000 adolescents aged 12 to 17 years 9 (2.7% of this age group) 10 had an AUD, with only 8.1% (18,000 males and 37,000 females) receiving treatment for an alcohol problem in a specialized facility.^11^

Excessive alcohol consumption accounts for nearly 88,000 deaths annually^4^ and is the fourth leading preventable cause of death in the U.S. 5 Alcohol-impaired driving fatalities account for 31% of overall driving fatalities.^12^ In addition, alcohol consumption contributes to non-fatal injuries resulting from traffic accidents, falls, and impaired judgment. Heavy alcohol drinkers suffer greater risk of alcohol dependence and withdrawal, liver cirrhosis and failure, and cancers of the mouth, esophagus, pharynx, larynx, liver, and breast.^13^–^15^ This high burden of alcohol-related injury and disease indicates a need to increase awareness of AUD and its effective treatment options.^8^

Given the rate of complications from AUD, the emergency department (ED) is a commonly used portal of entry into the healthcare system for many patients, and offers a unique opportunity for screening, brief intervention and referral to treatment (SBIRT).^16^, ^18^ Several professional and government organizations have already provided recommendations on implementation of SBIRT for certain patients, including those presenting with trauma.^19^, ^20^ However, little guidance exists on broader use of ED-based AUD interventions. This article provides a critical appraisal of the effectiveness of brief ED-based interventions as an injury-prevention strategy aimed at reducing alcohol intake and alcohol-related injuries among patients screened for AUD in the ED setting.

**Population Health Research Capsule**

What do we already know about this issue?

Screening, brief intervention, and referral to treatment (SBIRT) is an evidence-based practice used to identify substance abuse disorders, early intervention and treatment.

What was the research question?

What was the effectiveness of SBIRT at reducing alcohol intake for ED patients at risk for alcohol use disorder?

What was the major finding of the study?

Brief interventions in the ED showed a small reduction in alcohol use (low/moderate drinkers) and negative consequences.

How does this improve population health?

Alcohol use disorders and their negative consequences are a reason for ED visits, and any type of basic intervention may have an effect on subsequent outcomes of reducing harm in this population.

## METHODS

We conducted a systematic review of the literature regarding the effectiveness of SBIRT in the ED setting using the following key terms: alcohol consumption (related terms), alcohol reduction, alcohol dependence, alcohol screening, brief intervention, brief negotiated interview, computerized intervention, motivational interviewing, tailored feedback, injury, and emergency department. Electronic database searches of Medline (OVID), EMBASE (OVID), PsycInfo (OVID), The Cochrane Library (Wiley), CINAHL (EBSCO) and Web of Science (Databases: SCI-EXPANDED, SSCI, A&HCI) were conducted for English-language articles published between January 1966 and April 2016. We also considered websites of relevant organizations/networks and reference lists of included articles.

### Article selection and review

We selected articles for review based on information derived from the title, abstract, and keywords. If the title, abstract, and keywords did not yield enough information we then reviewed the full paper, We evaluated for inclusion all randomized studies of patients with known, suspected AUD, or alcohol-related injuries, assessing the effectiveness of brief ED-based interventions for the reduction of alcohol consumption, as well as the secondary goals of reducing alcohol-related negative consequences for both physical and social consequences of AUD,

Next, the articles that met inclusion criteria were appraised and assessed by two authors for their methodological quality, such as the method of randomization, blinding, allocation, description of withdrawals and dropouts, as well as loss to follow-up (Table).^18^ A third author reviewed the articles if there were any discrepancies in the grading. The reviewers were not blinded to the study hypothesis.

### Analysis

Given the lack of standardization across studies, including variations in patient populations, settings, screening techniques, and outcomes, data were analyzed descriptively. We focused on presenting trends and themes that emerged with regard to alcohol consumption and complications from continued alcohol use, such as injury. We also present the quality of studies that met our inclusion criteria.

## RESULTS

Thirty-five randomized control trials^21^–^55^ of patients of all ages seen in the ED with AUD were included (see Figure) in the final evidentiary table (Appendix A). The evidentiary table describes the target group, study design, primary and secondary outcomes, the main results, and the quality grading for each study.

Studies were generally limited to individuals older than 18 years with the exception of six studies that surveyed adolescents and young adults between the ages of 13 and 21 years old.^30^, ^35^, ^38^, ^41^, ^48^, ^49^

### Screening for Alcohol Use Disorder

The alcohol screening tools differed among the studies and included both self-reported questionnaires and biomarkers. Several structured questionnaires (Appendix B) were used to determine current and/or past alcohol use, and increased the sensitivity of self-report.^56^–^76^ Of the controlled randomized studies included in Appendix B, the self-reported screening instruments were as follows: one study used AUDIT (Alcohol Use Disorder Identification Test)-C^42^ and 12 studies used the full AUDIT.^23^, ^24^, ^28^, ^31^–^33^, ^38^, ^39^, ^42^, ^50^, ^53^, ^54^ Of these, 10 studies included all patients with a score of 8 or higher,^28^, ^31^–^33^, ^38^, ^39^, ^42^, ^50^, ^53^, ^54^ and two studies stratified the patients into three categories: low risk (0 to 6), at risk/moderate risk (7 to 18), and high risk (19 to 40). 3, 24 Authors mainly chose a lower cut-off score of 4 or greater for inclusion of adolescents.

In some studies, AUDIT was used with other alcohol screening tools such as the National Institute on Alcohol Abuse and Alcoholism (NIAAA) Guide,^31^ the CAGE questionnaire,^23^, ^39^ positive test for alcohol, and self-report of ingesting alcohol within six hours prior to the injury.^50^ Six studies^21^–^24^, ^35^, ^39^ used the CAGE questionnaire to screen injured patients for alcohol consumption; however, in one study it was followed by AUDIT to evaluate the quantity and frequency of alcohol consumption,^24^ while in another study the patients were initially screened using NIAAA followed by CAGE.^35^ Two studies, one in the United Kingdom and one in Australia, employed the Paddington Alcohol Test. (PAT)^29^, ^34^ *[PAT features a table of commonly encountered beverages coded in British units. Eight grams of alcohol are equivalent to one unit. The PAT allows for the different relative strengths of certain products, thus differentiating between a patient who may consume two pints (i.e., four “drinks”) of normal strength beer (four units) and the same amount of “strong” lager (10 units)].

Five studies used the NIAAA guide to screen patients.^21^, ^22^, ^31^, ^35^ In three of the five studies, NIAAA was used in conjunction with CAGE;^21^, ^22^, ^35^, in one additional study it was used with AUDIT, 31 and in another study it was used with Alcohol, Smoking and Substance Involvement Screening Test (ASSIST).^43^

Several instruments (Appendix C) were used to evaluate adolescent alcohol intake and consequences of drinking.^77^–^85^ Three studies involving adolescents 44, 48, 49 used Adolescent Drinking Questionnaire (ADQ) and Adolescent Drinking Index (ADI) instruments to evaluate alcohol consumption, and at follow-up used Adolescent Health Behavior Questionnaire and Short Michigan Alcoholism Screening Test (SMAST) to evaluate alcohol-related injuries. Eleven of the 35 studies used biomarkers (blood, breath or saliva tests) 25, 33, 36, 37, 39, 44–47, 48–50 as part of the screening tools.

### Instruments to Evaluate the Negative Consequences of Drinking Alcohol and Readiness to Change

Drinker’s Inventory of Lifetime Consequences (DrInC), a 45-item, self-report questionnaire about the negative consequences experienced from drinking that was validated on an alcohol treatment-seeking population of 1,728 inpatients and outpatients 86 and on Project MATCH, 87 was used by six studies 25, 27, 40, 50, 53, 55 to measure not only the physical but also the intrapersonal, social, interpersonal, and impulse control (e.g., driving while intoxicated, physical fights) consequences from drinking.

One author 50 used the Readiness to Change Contemplation Ladder 88 adapted for an ED treatment-seeking population of injured drinkers 69 to measure the subject’s attitude towards modifying alcohol-related behaviors with response categories ranging from 0 (no thought of changing) to 10 (taking action to change [e.g., cutting down]).

### Brief intervention and brief motivational intervention

Brief interventions (BI) are designed to motivate reduction and cessation of substance use by exploring and highlighting individual risks and negative outcomes of problematic substance use. Though it is not intended to treat people with serious substance use disorders, it can be used to encourage those with more serious dependence to accept either more intensive treatment within the primary care setting or a referral to a specialized alcohol and drug treatment agency.

The most common behavioral therapies used in SBIRT programs are brief versions of cognitive behavioral therapy and motivational interviewing or some combination of the two. Brief interventions can be made more effective by using the technique of motivational interviewing. The principles of brief motivational interviewing (BMI) and asking for permission to discuss alcohol use; (2) providing feedback on current drinking and consequences; (3) assessing readiness to change; and (4) providing options to help with behavioral changes and assisting in obtaining appointments or placements if desired.^91^

ED-based brief interventions were performed by a variety of professionals and staff members including, physicians, medical students, mid-level providers,^21^, ^31^ nurses,^35^ social workers, psychologists,^23^, ^36^, community outreach workers and “health promotion advocates.”^24^ ED staff nurses trained to conduct SBIRT were less fully engaged with SBIRT implementation when the ED was extremely busy. 35 The training required to prepare staff for delivering BI included reading review of materials about the assessment of adverse consequences of alcohol abuse, 89 as well as structured sessions to teach and practice the principles and techniques of SBIRT. 20

### Main Outcomes

All studies used reduction of alcohol consumption as the primary outcome measure. Thirteen studies (37%) enrolling a total of 5,261 participants reported significant differences between control and intervention groups defined by the number of drink days and number of units per drink day.^21^–^23^, ^28^, ^29^, ^36^, ^37^, ^39^, ^40^, ^44^, ^45^, ^50^, ^51^ Sixteen studies (46%) showed a reduction in alcohol consumption in both the control and intervention groups. 25, 26, 31– 37, 42, 43, 47, 49, 50, 52, 55 Nine^25^, ^26^, ^35^–^37^, ^42^, ^49^, ^50^, ^55^ out of these 16 studies showed greater improvement in the BI group as compared to the control group as follows: a higher reduction in the overall consumption of alcohol;^25^, ^26^, ^35^, ^49^ reduction in the concomitant use of marijuana and alcohol;^55^ and fewer injuries.^35^, ^36^, ^50^ However, the effectiveness of the interventions in reducing at-risk drinking was weakened at six- and 12-month follow-up points.^22^, ^29^–^33^, ^41^, ^46^, ^48^, ^49^

Seventeen out of 35 studies failed to demonstrate an intervention effect for the primary outcome of alcohol consumption reduction.^21^, ^24^–^27^, ^30^–^35^, ^38^, ^41^–^43^, ^46^–^49^, ^51^–^55^ However, 11 of those 17 studies (65%) enrolling a total of 4,706 participants observed some significant differences between BI and control conditions, at least for specific subgroups or secondary outcome criteria.^21^–^26^, ^38^, ^41^, ^42^, ^47^, ^48^, ^53^–^55^ For example, one author found^24^ statistically significant changes in “trying to be careful while drinking” in the intervention group in patients 18–21 years old with low AUDIT scores, and another^26^ reported decrease in drinking, drinking days per week, maximum drinks per occasion and negative consequences of drinking in injured patients older than 18 years old. Among adolescents, a subgroup with a history of previous drinking and driving, the intervention group showed a beneficial effect in the reduction of drinking and driving.^41^ Additionally, Segatto^47^ found in adolescent and young adult patients a decrease in the following outcomes: days of alcohol use; days with moderate and heavy use; and negative consequences. Spirito et al.^48^ found that the subgroup of adolescents who screened positive for problematic alcohol use at baseline reported significantly more improvement with fewer drinking days as well as fewer high-volume drinking days. Focusing on women, for example, the subgroup age 22 years, a reduction was found on the Drinker Inventory of Consequences (DrInC) in the intervention group.^25^ Havard et al.^38^ also found that women in the intervention group engaged in heavy drinking at one third of the frequency as the control group.

In some studies BI was shown to have an effect only on low or moderate drinkers 23 and not on high-risk or dependent drinkers (defined as an AUDIT score >15 or >18, respectively). However, Mello et al.^42^ found the subgroup of participants with AUDIT scores>15 in the BI group had a lower three-month impaired score. If the participants attributed their injury to alcohol, Walton et al.^53^ demonstrated lower levels of average alcohol consumption and less-frequent heavy drinking in the BI group. In addition, Wang et al.^54^ found a significant increase in readiness to change in the BI group (in excessive alcohol users, AUDIT 2+ for men and 1+ for women), but not in the control group. Woolard et al.^55^ showed binge drinking and concomitant marijuana use decreased for the BI group.

### Readiness to Change Combined with BMI

A study by Stein 50 looked at pretreatment readiness to reduce drinking as a mediator of BMI effectiveness on alcohol-related consequences and found positive effects only on those highly motivated to change prior to the intervention but not for those with low pre-intervention motivation.

### ED Referral to Outpatient Alcohol Health Worker

In the United Kingdom study by Crawford, 29 the patients were screened in the ED and then referred for outpatient follow-up with an alcohol health worker (AHW) for about 30 minutes of assessment and discussion of current and previous drinking. Of those referred, 65.8% followed up with an AHW. Alcohol consumption in patients who followed up with an AHW decreased to a mean of 59.7 units* per week as compared with 83.1 units in patients in the control group (t –2.4, p=0.02). At 12 months, those who pursued follow-up were drinking 57.2 units per week compared with 70.8 in controls (t –1.7, p=0.09). This study also showed that the patients followed by the AHW had a mean of 0.5 fewer visits to the ED over the following 12 months (1.2 compared with 1.7, t –2.0, p=0.046).

## DISCUSSION

The studies reviewed employed several alcohol screening tools, including in order of frequency the AUDIT, CAGE, NIAAA, and PAT. Although longer than other tools, the AUDIT can be completed in one minute and 13 seconds and its test characteristics make it preferable in study settings.^58^ However, AUDIT-C, CAGE and MAST are designed for a range of health settings and are particularly appropriate for use in the ED because of their brevity and their focus on harmful drinking.^90^ Despite these validated and easily applied tools the minority of patients (less than one in five) ever reports being questioned by physicians about alcohol use. 93

Most studies employed a face-to-face BI delivered by healthcare personnel (nurses, doctors, or social workers) who had received specialized training. A few studies used booster sessions delivered after the initial BI. There was no difference in short-term and long-term outcomes in the studies that used one session as compared to studies that had a follow-up BI session.

All studies used reduction of alcohol consumption as the primary outcome. Many studies showed an improvement in AUD in both the control and intervention group. Our interpretation of the data from these studies suggests that the simple intervention of a doctor showing concern while questioning a patient’s excessive alcohol consumption reinforces the connection between drinking and the patient’s health issues. This brief intervention alone, provided in most of the control groups, goes beyond what most providers do in current practice and is likely to be effective in decreasing patients’ harmful drinking as reflected by the reduction in alcohol consumption seen in the control patients in our reviewed studies. More intensive and costly interventions had limited additional benefit beyond the control group efforts. In reality, the minimal effort provided for these control groups amount to a significant intervention over baseline practices and should be considered for inclusion during all patient encounters. Since AUD and alcohol-related problems are a frequent reason for ED visits, any type of basic intervention implemented in the ED itself may have an important effect on subsequent outcomes of potentially reducing harm in this population.

Future areas of focus will need to look more closely at subpopulations identified by their willingness/readiness to change. Targeted interventions are more likely to have benefit, and scale-up of such interventions requires judicious use of resources in busy EDs. In addition, future studies will need to more closely examine the duration of BI/BMI effect. This information would allow for a more evidence-based approach to determine the need and frequency for booster sessions as a tool for maintaining long-term outcomes and sustainability.

## LIMITATIONS

This systematic review included a heterogeneous group of studies; most of the studies were conducted in the U.S., with one study from the UK and one from Australia. We only included trials published in English. The abstractors were not blinded to the study hypothesis. We did not conduct a formal meta-analysis of the trials identified.

## CONCLUSIONS

Among adults and children 12 years of age and older, the effectiveness of BI/BMI during an ED visit for alcohol use-related problems has been inconclusive, with heterogeneity of conditions and outcomes researched across studies. Nevertheless, a small but important number of studies have demonstrated small reductions in alcohol consumption, in negative consequences of alcohol use (such as injury), and in ED repeat visits. In addition, BI/BMI delivered in the ED appears to have at least short-term effectiveness in reducing at-risk drinking, possibly highlighting the need for supplementing the ED-based BI/BMI with referrals to outpatient programs equipped to maintain long-term contact with risky drinkers to sustain its effect. Although there are challenges to universal implementation of BI/BMI in the ED, the positive effect of asking about alcohol consumption seen in control groups is heartening in that relatively low-intensity intervention strategies may help our patients reduce the harmful effects of alcohol consumption.

## Supplementary Information







## Figures and Tables

**Figure:**
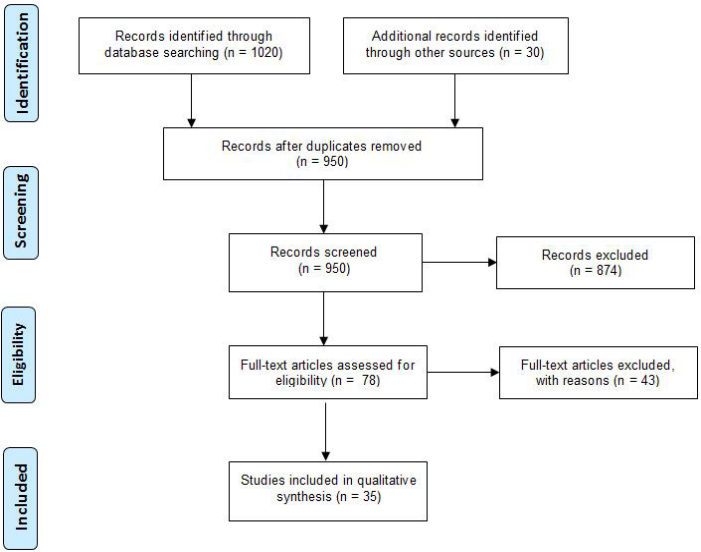
PRISM flow diagram^94^ for a systematic survey of studies that looked at the effectiveness of brief interventions in emergency department patients with suspected alcohol use disorder.

**Table:** Scoring system used in a survey looking at the effectiveness of brief interventions for suspected alcohol use disorder.

Type of study	Question	Score
Randomized control trials	Was the study described as randomized?	1 for “yes” 0 for “no”
	Was the study described as double blind?	1 for “yes” 0 for “no”
	Was there a description of withdrawals and dropouts?	1 for “yes” 0 for “no”
	a) Was the method to generate the sequence of randomization described and was it appropriate (random numbers, computer generated, etc.)?	1 for “yes” 0 for “no”
	b) Was it inappropriate (alternate allocation, by date of birth, chart number, etc.)?	−1 for “yes” 0 for “no”
	a) Was the method of double blinding described and appropriate (identical placebo, etc.)?	1 for “yes” 0 for “no”
	b) Was it inappropriate (comparison of tablet to injection without double dummy, etc.)?	−1 for “yes” 0 for “no”
	Was the loss to follow-up rate greater than 20%?	−1 for “yes” 0 for “no”
