# Supplementary material for: Effectiveness of SBIRT for Alcohol Use Disorders in the Emergency Department: A Systematic Review
Source: West J Emerg Med. 2017 Sep 21;18(6):1143–52. doi: 10.5811/westjem.2017.7.34373 (PMC5654886; doi:10.5811/westjem.2017.7.34373)
Supplement: Supplementary file 1 [file wjem-18-1143-s001.docx]

Appendix A. Evidentiary Table.

| Authors | Target Group | Design and Methods | Intervention Details | Outcome Measures:  Tools/Follow up | Main Results | Comments/Grading |
| --- | --- | --- | --- | --- | --- | --- |
| The Academic ED SBIRT Research Collaborative Group (Authors: Aseltine RH, Bernstein E, Bernstein J, et al.) *Ann Emerg Med*. 2007;50(6):699-710. USA | Injured patients treated in ED, 14 academic EDs across the USA, quasi-experimental comparison group design,18+ years, both genders; screened positive for drinking over the NIAAA National Institute of Alcohol Abuse and Alcoholism low‐risk limits  (N = 1,132) | BI (551), CG (581), patients who screened over the low‐risk limits were asked to participate. | BI: Trained staff delivered the Brief Negotiated Interview. The mean time for completion was 7.8 minutes, with a range of 4 to 24 minutes.  Patients received also a written handout.  CG: written handout only. | Self‐report form, baseline questions including basic demographic and socioeconomic information.  Follow Up: 3 months (62%), telephone interviews; same as baseline, participation in and completion of any other alcohol treatment options. | 3‐month follow‐up: BI group reported significantly lower levels of drinks per week and maximum drinks per occasion. 3‐month decrease in number of weekly drinks by 37% in BI group (22.5 to 14.1) and 24% in CG (22.9 to 17.4), in maximum number of drinks by 24% in BI group (8.6 to 6.5) and 14% in CG (8.4 to 7.2). 28% of BI group and 18% of CG no longer exceeded low‐risk limits.  No differences between the groups concerning participation or completion of any other alcohol treatment. | Grade 3/5 |
| The Academic ED SBIRT Research Collaborative Group (Authors: Aseltine RH, Bernstein E, Bernstein J, et al.) *Alcohol Alcohol.* 2010;45(6):514–519. USA | As above | As above | As above | 3 months (62%), 6 months (52%), 12 months (38%), telephone interviews; same as baseline, participation in and completion of any other alcohol treatment options. | Effects at 3 months had weakened and were no longer statistically significant at 6 and 12 months. | Grade 3/5 |
| Bazargan‐Hejazi S, Bing E, Bazargan M, et al. *Ann Emerg Med.* 2005;46(1):67-76. USA | Injured patients treated in ED; 18+ years, both genders; CAGE score ≥ 1; (N = 295) | BI (151), CG (144); after written consent, patients were screened for at‐risk drinking, screen positive (CAGE ≥ 1) were randomized. | BI: trained Health Promotion Advocates from community‐based organizations delivered a 15- to 20‐minute structured intervention—the semi-scripted Brief Negotiated Interview developed by Rollnick, Bernstein, and Bernstein.  CG and BI: received a "health packet" with  a variety of health information | CAGE, AUDIT, structured questionnaire included readiness to change drinking behavior, and readiness to enter alcohol treatment;  Follow Up:  3 months (63%), face‐to‐face in ED, same as baseline. | Patients were divided into 3 groups depending on AUDIT scores: low‐risk (0 to 6), at‐risk/moderate‐risk (7‐18), high‐risk (19‐40).  Reduced their risk (moved to a lower risk category): BI (48%), CG (38%)  At‐risk/moderate group (reduced risk): BI (34%), CG (13%)  High‐risk group (reduced risk): BI (66%), CG (60%)  The only significant predictors of outcome were intervention group  status and baseline AUDIT. | Grade 2/5 |

| Authors | Target Group | Design and Methods | Intervention Details | Outcome Measures:  Tools/Follow up | Main Results | Comments/Grading |
| --- | --- | --- | --- | --- | --- | --- |
| Bernstein J, Heeren T, Edward E, et al. *Acad Emerg Med.* 2010;17(8):890–902. USA | Injured and non-injured (intoxicated) patients treated in Level 1 TC (PED) TC, 14‐21 years, both genders;  5+ drinks in 2 hours for males and 4+ drinks in 2 hours for females, or high‐risk behaviors in conjunction with alcohol use, and/or AUDIT score 4+ for ages 14‐17 years or 8+ for ages 18‐21 years  (N = 853) | BI (283), CG‐Assessment (284), CG (286); patients screened positive were asked to participate, consent obtained from parents or guardian (written or oral) | BI: trained peer educators younger than 25 years (Bachelor degrees) delivered 1 20‐ to 30-minute MI session and a 10‐day, 5‐ to 10‐minute booster telephone call.  CG‐A: same assessment as BI and written advice about alcohol‐related risks (Several BI sessions) | TLFB, AIC, Drinking and driving scale, AHBQ, PHQ‐A depression scale, PTSD Checklist Civilian version (length NR).  Follow Up: 3 months (70%), BI and CG‐A only, 12 months (72%) BI, CG‐A, CG; same as assessment, including abstinence at 12 months (follow‐up visits in ED) | No statistically significant differences between BI and CG‐A in alcohol consumption (drinking days per months, mean drinks per drinking day, mean drinks per week, maximum drinks per day) and alcohol-related consequences.  A significantly larger proportion of the BI group made efforts to change consumption and drinking behaviors.  73% (BI) vs. 65% (CG‐A) tried to cut back on drinking, 41% (BI) vs. 28% (CG‐A) tried to quit drinking, 81% (BI) vs. 71% (BIA) tried to be careful when drinking.  A statistically significant group effect could be found for ages 18‐21 years (not for ages 14‐17 years), for those with low AUDIT scores (below the cut point for hazardous drinking) except "tried to be careful" (not for those with high AUDIT scores), for those with post-traumatic stress disorder (tried to quit drinking) and without post-traumatic stress disorder (tried to cut back and tried to be careful). | Grade 3/5 |

| Authors | Target Group | Design and Methods | Intervention Details | Outcome Measures:  Tools/Follow up | Main Results | Comments/Grading |
| --- | --- | --- | --- | --- | --- | --- |
| Blow FC, Barry KL, Walton MA, et al. *J Stud Alcohol.* 2006;67:568–578. USA | Injury patients treated in Level 1 TC ED, 19+ years, both genders;  BAC 100‐200 mg/dl (N =494) | TM‐BI: tailored message, BI (129), TM‐NoBI: tailored message. no BI (121), GM‐BI: generic message, BI (124), GM‐NoBI: generic message, no BI (120); BAC level was assessed during ED visit, if BAC 100‐200 mg/gl, patients were screened with a computerized health survey on a desktop computer | TM‐BI and TmNoBI: computer generated tailored message, GM‐BI and GMNoBI: computer-generated generic massage. The feedback consisted of a booklet printed by the computer for each participant. The booklets were identical in length, content, and graphics and included either messages tailored to the individual responses or standard messages.  For the advice conditions (TM‐BI and GM‐BI), a research social worker conducted a BI‐session (duration NR), focusing on reviewing the booklet using the FRAMES approach. For the no‐advice conditions, patients were given the booklet without a BI‐session. | Alcohol‐related questions embedded in lifestyle screening survey, SDrInC, with two additional items from the longer DrInC (arrested for DUI, hurt, injured, or burned while intoxicated)  Follow Up: 3 months (85%), 12 months(85%), telephone contact, same as baseline (Patients received $20 at 3 months and $30 at 12 months) | AT 12 months, all four groups had significantly reduced the weekly intake with highest reduction in TM‐BI group (TM‐BI: 49%, TM‐NoBI: 26%, GM‐BI: 35%, GM‐NoBI: 36%).  At 12 months, all four groups had a significantly reduction in HED (TM‐BI: 24%, TM‐NoBI: 20%, GM‐BI: 36%, GM‐NoBI: 37%).  Women age ≤22 years who received advice were the most likely to decrease HED.  The decrease in scores on the DrInC was significant in all of the four groups (TM‐BI: 32%, TM‐NoBI: 26%, GM‐BI: 16%, GM‐NoBI: 31%).  Women age ≤22 years who received advice were the most likely to show a reduction on the DrInC. | Grade 3/5 |
| Cherpitel CJ, Moskalewicz J, Swiatkiewicz G, et al. *J Stud Alcohol Drugs.* 2009;70(6):982-990. Poland | Injured patients treated in ED, 18+ years, both genders; screened positive if answers were positive on any one of the RAPS4 items, or for men: 11+ drinks/week or 4+ drinks/occasion; for women: 6+ drinks/week or 3+ drinks/occasion (lower than NIAAA guidance) (N = 446) | BI (145), CG Assessment (152), CG‐Screening (147); patients screened positive were asked to participate. | BI: Trained nurses delivered 1 15- to20‐minute Brief Negotiated Interview.  CG‐Assessment (CG‐A): same assessment as BI (Several BI sessions were audiotaped.) | BAC, TLFB, SIP, DrInC, Readiness to Change Ruler, Risk taking/impulsivity adapted from Eysenk  Follow Up: 3 months (84%) BI and CG Assessment only TLFB, SIP, Readiness to Change Ruler, participation in any other alcohol treatment options (telephone contacts or in person) | At 3‐month follow‐up no significant differences in outcome measures between BI and CG‐Assessment conditions.  Significant decrease of at‐risk drinking (BI=27%, CG‐A=34%), of drinking days per week (BI=28%, CG‐A=26%), of drinks per drinking day (BI=24%, CG‐A=26%), of maximum drinks on an occasion (BI=25%, CG‐A=20%), and of negative consequences (BI=56%, CG‐A=46%). | Grade 3/5 |

| Authors | Target Group | Design and Methods | Intervention Details | Outcome Measures:  Tools/Follow up | Main Results | Comments/Grading |
| --- | --- | --- | --- | --- | --- | --- |
| Cherpitel CJ, Korcha RA, Moskalewicz, J, et al. *Alcohol Clin Exp Res.* 2010;34(11):1922-1128. Poland | same | same | same | 12 months (63%), BI and CG Assessment only and (63%) Screening group | At 12‐month follow‐up at‐risk drinking and number of drinks per drinking day decreased for the 3 conditions.  At‐risk drinking: BI=27%, CG‐A=27%, CG=38% number of drinks per drinking day: BI=27%, CG‐A=21%, CG=30% Significant declines of RAPS4 (44%), number of drinking days per week (28%), and maximum number of drinks on an occasion (29%) were only seen for the intervention condition. Significant decrease of negative consequences for BI (70%) and CG‐A (47%). | Grade 3/5 |
| Cobain K, Owens L, Kolamunnage‐Dona R, et al. *Alcohol Alcohol.* 2011;46(4):434-440. UK | Injured patients treated in ED, 18+ years, both genders; AUDIT 16+ and SADQ 1+ (positive) (N = 200) | BI (100), CG (100); prospective cohort control study, test site was a university hospital ED, the control site a district general hospital (ED).  Screened positive (AUDIT, SADQ) were asked to participate. | BI: a trained Alcohol Specialist  Nurse (ASN) delivered 15‐ to 20‐minute sessions. "Nurses made a clinical judgment of how many times a further follow‐up appointment should be offered." Median of 3 20‐ minute sessions.  CG: normal clinical care. | AUDIT, SADQ Follow Up: 6 months (49%); AUDIT, SADQ either by telephone or face‐to‐face by research nurse | At 6 months statistically significant difference between BI group and CG concerning AUDIT score, SADQ, number of drink days, and number of units per drink day, in BI group 37% reported total abstinence (CG 0%), severity of alcohol dependence was reduced in 77% (BI group) and 20% (CG) | Grade 2/5 |
| Crawford MJ, Patton R, Touquet R, et al. *Lancet.* 2004;364(9442):1334-1339. UK | Injured patients treated in ED, 18+ years, both genders; PAT positive (misusing alcohol) (N = 599) | BI (287), CG (312); Screened PAT positive (part of routine practice) were asked to participate | BI: trained nurses (alcohol health workers) delivered 1 30‐minute session;  CG: were given a health information leaflet | PAT, demographic and clinical details collected as part of routine assessment  Follow up: 6 months (61%), 12 months (64%); 6‐months: PAT, 90‐AQ, General Health Questionaire; 12‐months: PAT, 90‐AQ, Time Line Follow Back (Steady Pattern Grid), EQ‐5D | 6‐month and 12‐month follow‐up. Mean units consumed during drinking session (SD): At 6‐month follow‐up decrease from 21.5 to 13.0 (BI) and 20.9 to 17.1, relatively stable after 12 months (31.1 BI; 16.0 CG).  Mean units of alcohol per week: At 6‐month follow‐up BI‐group drank fewer mean units (59.7) than CG (83.1), after 12 months those in BI drank less than those in CG (57.2 vs. 70.8), difference no longer significant (no baseline data presented). | Lower levels of alcohol consumption in BI were not associated with differences in mental health or quality of life. CG may benefit from the health information leaflet.  Grade 3/5 |

| Authors | Target Group | Design and Methods | Intervention Details | Outcome Measures:  Tools/Follow up | Main Results | Comments/Grading |
| --- | --- | --- | --- | --- | --- | --- |
| Cunningham RM, Chermack S T, Ehrlich PF, Carter PM, Booth BM, Blow FC, Walton MA. Alcohol interventions among underage drinkers in the ED: a randomized controlled trial. *Pediatrics*, 2015; *136*(4), e783-e793. USA | 14-20 yrs. old drinkers  Level 1 TC ED;  (AUDIT-C;  Age 14–17 years, score 3+; age 18–20 yrs., score 4+)  (N = 836) | Randomized to 1 of 3  ED-based conditions (computer BI,  therapist BI, or control) and 1 of 2  booster conditions (post-ED session  or control) that were administered after the 3-month follow-up interview.  Self-administered the 15- to 20-minute survey via touchscreen tablet received a $20 gift; Remuneration was $35 for 3-month follow-up, $35 for 6-month follow up, and $45 for 12-month follow-up. | Computer BI: touchscreen tablets;  Therapy BI;  CG: brochure;  At follow up a BI delivered by a therapist blinded to ED condition assignment or no BI  (control) | Primary Outcomes: alcohol consumption index and alcohol consequences using the18-item RAPI; secondary outcomes: driving under influence using the 5-item Young Adult Driving Questions; alcohol related injury; and drug use.  Follow up: 3-month, 6 and 12 interviews. | Primary Outcome:  At 3 months: Computer BI and therapist BI significantly decreased the alcohol consumption index score and alcohol consequences compared with control; while at 12 months: only consequences.  Secondary Outcomes: At 12 months computer BI reduced the frequency of DUI  and prescription drug; Therapist BI reduced the frequency of alcohol-related injury at 12 months.  The post-ED session reduced alcohol consequences at 6 months, benefiting those who had not received a BI in the ED.  Follow-up rate: 86.8%  at 3 months, 87.1% at 6 months, and 88.0% at 12 months | 4/5 |
| D´Onofrio G, Pantalon MV, Degutis LC, et al. *Ann Emerg Med.* 2008;51(6):742-750. USA | Injured patients treated in ED, 18+ years, both genders; screened positive for drinking over the National Institute of Alcohol Abuse and Alcoholism  (NIAAA) low‐risk limits or presenting in ED with an injury associated with alcohol use, and AUDIT score 19 or less.  (N = 500) | BI (250), CG (250); patients who screened over the low‐risk limits were asked to participate. | BI: trained emergency practitioners delivered a 10‐minute structured intervention—the semi-scripted Brief Negotiated Interview.  CG: trained emergency practitioners read scripted discharge instructions, 1 minute. A handout with more information was provided. (all assess treatment, BI and CG, were audiotaped) | NIAAA Guidelines, AUDIT, TLFB, health screening questions, drinking‐related consequences, the Contemplation Ladder, Short Form Health Survey, Treatment Service Review  Follow Up: 6 months (95%), 12 months (92%), telephone contacts, probably same as baseline (not reported in details) | At 6 months and 12 months no differences between BI group and CG concerning all outcome variables (HED, average number of drinks per week, negative consequences, occurrence of injuries, treatment service utilization).  All groups showed statistically significant reduction in average number of drinks per week, HED and proportion over NIAAA Guidelines.  Mean number of drinks per week: At 12‐month follow‐up decrease from 13.6 to 3.8 (BI) and 12.4 to 2.6 (CG). HED: At 12‐month follow‐up decrease from 6.0 to 2.0 (BI) and 5.4 to 1.5 (CG). Proportion over NIAAA Guidelines: At 12‐month follow‐up decrease from 99.2% to 62.0% (BI) and 98.0% to 65.4% (CG). | CG also received brief advice, (with 62% [66%] of patients with AUDIT score below 8 enrolled it is difficult to demonstrate improvement);  Grade 3/5 |

| Authors | Target Group | Design and Methods | Intervention Details | Outcome Measures:  Tools/Follow up | Main Results | Comments/Grading |
| --- | --- | --- | --- | --- | --- | --- |
| Daeppen JB, Gaume J, Bady P, et al.  *Addiction.*2007;102(8):1224-1233. Switzerland | Injured patients treated in ED, 18+ years, both genders; hazardous drinkers were defined as 14+ drinks per week or 5+ drinks on one occasion for men in the past 30 days or 7+ drinks per week or 4+ drinks on one occasion for women or men over 65 years.  (N= 987) | BI (310), CG with assessment (342), CG without assessment (335); complete a 2‐minute lifestyle screening survey; screen positives were asked to participate. | BI: Research assistant (master´s level psychologist or experienced ED nurse) conducted 1 30‐minute assessment after screening, then delivered 1 15‐ minute MI.  CG with assessment: same assessment as BI  CG without assessment: no assessment | Alcohol‐related questions embedded in lifestyle survey (drinks per week, amount per drinking day, frequency of HED).  Assessment includes AUDIT, SF‐12, TLFB past 7 days;  Follow up: 12 months (78%), telephone interviews; same as baseline | No differences between the three groups. All groups showed similar reductions in drinking frequency, quantity, frequency of HED, and AUDIT scores at 12 months. Changed to low‐risk drinking: 36% (BI), 34% (CG with assessment), 37% (CG without assessment)  BI too short, decrease may be attributed to regression to the mean | 3/5 |

| Authors | Target Group | Design and Methods | Intervention Details | Outcome Measures:  Tools/Follow up | Main Results | Comments/Grading |
| --- | --- | --- | --- | --- | --- | --- |
| Dauer AR, Rubio ES, Coris ME, et al. *Alcohol Alcohol*. 2006;41(1):76-83. Spain | Motor vehicle (drivers, passengers, pedestrians) injury patients treated in Level 1 TC ED, 18+ years, both genders, positive alcohol test, BAC 0.2+ g/L  (N = 85) | BI (40), BI‐CG (45) Minimal intervention; routine screening in ED, screen positive (BAC ≥ 0.2 g/L) were asked to participate | BI: trained nurses and social work staff delivered 1 15‐ to  20‐minute.  Patients were given a leaflet (same as CG‐BI) and a self‐help leaflet.  CG‐BI: trained nurses and social work staff delivered 1 5‐minute "minimal intervention" limited to empathic advise, and a leaflet. | AUDIT, AUDIT‐C, AIS, The Readiness Ruler, ad hoc inventory exploring behaviors (eg, drinking before event, other traffic crashes)  Follow Up: 3 months (67%), 6 months (60%), 12 months (67%) telephone contacts, AUDIT‐C, at 12 months frequency of traffic accidents in the study period | No significant differences between the two treatment groups for all parameters. Both groups profit from the interventions. At 12 months (ITT analysis), 47% had reduced their consumption, 35.2% had ceased drinking at hazardous level, 50% of former AUDIT‐C positive patients had become negative.  The amount of reduction was larger among patients who scored positive in AUDIT and/or AUDIT‐C than (those who scored) negative.  60% drop in accident rate during the follow‐up period compared to the year prior to the study entrance. | Authors speculated on reasons for lack of differences between the two treatment groups: a deterrence effect of the crash, assessment after the injury, regression‐to‐mean, chance, and the possibility, that any intervention after such aversive events may have an effect.  Grade 2/5 |
| Dent AW, Weiland TJ, Phillips GA, et al. *Emerg Med Australas.* 2008;20(2):121-128. Australia | Injured patients treated in ED, 18+ years, both genders; PAT positive (misusing alcohol)  (N = 468) | BI in ED (159, 149 received BI), BI‐MI after discharge (148, 15 received MI), CG (161); Screened PAT positive (part of routine practice) were asked to participate, group allocation was not blinded to the patients. | BI in ED: trained doctor or nurse delivered a semi-scripted informative discussion. A letter reinforcing the information discussed was provided. Median duration was 5 minutes.  BI‐MI: trained doctor or nurse delivered MI within 1 week at ED. Median duration was 45 minutes.  CG: standard care. | PAT Follow Up: 1 month (62%), 3 months (54%); PAT, number of re-presentations to any ED | In the ITT‐analysis each group had statistically significant reduced the alcohol intake. BI and BI‐MI groups demonstrated no advantage over CG. | Life‐threatening injury may have affected the general outcome, low follow‐up rates.  Grade 2/5 |
| Désy PM, Howard PK, Perhats C, et al. *J Emerg Nurs.* 2010;36(6):538-545. USA | Level 1 TC ED with injuries that were not life-threatening; screened positive for at-risk or unhealthy drinking  N=91 | BI (49); CG (42) resources for follow-up care and community services appropriate to their alcohol use risk level. | BI: Staff nurses were trained to conduct SBIRT. At-risk drinking: BI 5 to 10 minutes to motivate them to cut back or quit drinking. Educational brochures and a list of community resources. All abstainers, including those who rarely drank low amounts of alcohol, were submitted to standard care procedures. | NIAAA quantity and frequency questions and the CAGE questionnaire. The primary outcome variables of interest were alcohol consumption (number of drinks and drinking days per week), recurring ED visits, compliance with referrals, and traffic violations and crashes.  Follow Up: 3 months 50.5% | Decline in alcohol consumption in the intervention group was significant (28.6 to 8.0 drinks per week), the difference between the mean number of drinks for the intervention and usual care groups at follow up (8.0 vs. 7.7 per week) was not statistically significant.  BI (20%) had recurring ED visits compared with patients in the CG (31%) during the 3-month follow-up period, but the difference between the 2 groups was not statistically significant. | Patients in the BI had more than a 10% decrease in alcohol consumption and 5% fewer alcohol-related injuries during the follow-up period compared with patients in the usual care group. |
| Authors | Target Group | Design and Methods | Intervention Details | Outcome Measures:  Tools/Follow up | Main Results | Comments/Grading |
| Field CA, Caetano R. *Drug Alcohol Depend.* 2010;111(1-2):13-20. USA | Injured patients treated in Level 1TC ED, 18+ years, both genders; BMI vs. TAU+. One of 4 criteria: positive BAC or self-reported drinking within 6 hours before injury, drinking pattern beyond NIAAA standards NIAAA guidelines (eg, 7 drinks/week women, 14 drinks/week men; more than 4 drinks/day in men; more than 3 drinks/day in women; NIAAA, 2005) or + on 1 or more items of the CAGE. (N=1,336) | Injured patients: black, white or Hispanic who screened positive on one or more criteria were asked to participate. (compensation $25 for the baseline assessment and $50 for the 6- and 12-month follow-up assessments).  BI Training extensive: didactic lectures, video examples and role play, reading first 11 chapters of Preparing People for Change by Miller and Rollnick (2002); 3 days of training by experienced trainer in motivational interviewing. Two days of training in the trauma care setting. | BI: Trained MPH and licensed professional counselors BI 30–40 minutes. Alcohol use, alcohol problems and dependence status among injured patients meeting criteria for alcohol dependence during baseline assessment.  CG: TAU+ given handouts.  10% of interventions were randomly selected to be audio taped. | NIAAA, CAGE and 1) alcoholics anonymous, 2) alcohol detox program, 3) alcohol recovery home or residential program, 4) outpatient program, 5) drinking and driving program or other mandated education, or 6) other type of services for alcohol problems (patients were compensated).  Blinded Follow up by telephone at 6 and 12 months: 6 months 77%; 12 months 66%. Hispanics were less likely to complete 6-month follow up. | 668 whites (45%), 537 Hispanics (36%) and 288 (19%) blacks. BMI: significantly higher reductions in volume per week at 6- and 12-month follow up and significant decreases at twelve months in percent days abstinent (β =.11, p=.007) and alcohol problems (β =− 2.7, p12 =.04). Among patients with alcohol dependence .59 (95% CI=.39–.91) times less likely to meet criteria for alcohol dependence at 6 months. | BMI is more beneficial among patients with alcohol dependence who screen positive for an alcohol related injury.  Grade 3/5 |
| Field CA, Caetano R, Harris TR, et al. *Addiction.* 2010;105(1):62-73. | Same as above; | Same as above;  Hierarchical linear modeling to evaluate ethnic differences in drinking outcomes including volume per week, maximum amount consumed in one day, percent days abstinent, and percent days heavy drinking at 6- and 12-month follow up. | Same as above | Same as above | BMI: at 6- and 12-month follow up, Hispanics significantly reduced maximum amount consumed in one day (p<.001; p<.001, respectively) and percent days heavy drinking (p<.05; p<.05, respectively). At 12 months, Hispanics reduced average volume per week (X2 =6.8, df=1, p<.01). TAU+: at 6- and 12-month follow up, Hispanics reduced maximum amount consumed (p<.001; p<.001); 12-month follow up and volume per week at (p<.001).  Whites and blacks in both BMI and TAU+ reduced volume per week and percent days heavy drinking at 12-month follow up (p<.001; p<.01, respectively) and decreased maximum amount at 6- (p<.001) and 12-month follow up (p<.001). | Grade 3/5 |
| Authors | Target Group | Design and Methods | Intervention Details | Outcome Measures:  Tools/Follow up | Main Results | Comments/Grading |
| Havard A, Shakeshaft AP, Conigrave KM, et al. *Alcohol Clin Exp Res.* 2012;36(3):523–531. Australia | Injured patients treated in ED,14+ years, both genders; AUDIT score 8+ (N = 304) | BI (150), CG (120); patients identified as risky drinkers (AUDIT score 8+) were asked to participate in follow‐up surveys. | BI: A personalized normative feedback letter was mailed to the participants.  CG: no intervention | Modified AUDIT only screening  Follow up: 6 weeks (80%); modified AUDIT, by telephone, e‐mail, or mail. | At 6 weeks, no significant effect of BI was observed. Patients with alcohol‐involved ED presentation consumed fewer drinks per week in BI group (11.9) than in CG (24.1) at 6 weeks. At follow‐up, women in BI group  engaged in heavy drinking at one‐third the frequency of women in CG (1.6 vs. 4.5), PP analysis. |  |
| Kunz FM Jr, French MT, Bazargan-Hejazi S. *Stud Alcohol.* 2004;65(3):363-370. USA | ED at least 18 years old (29), present in the ED to receive medical care (57), be able to speak either English or Spanish (0), have used alcohol in the past 12 months (433), have answered at least one of four CAGE questions affirmatively and not have received alcohol counseling in the past year (29).  N=294 | CAGE and incidence of alcohol-related injuries and violence, alcohol and drug use and types of drinks consumed. Baseline ($10) and follow up ($20) surveys. | Health promotion advocates (HPAs). Those “not ready” agreed to seek more information about drinking; those “unsure” were guided to think more about the negative consequences of drinking; and those “ready” agreed to lower their drinking per day, per week and per occasion.  BI: brief counseling session; received health information packet, a copy of their action plan, and a reminder card for a follow-up session.  CG: participants received only the packet of health information. | AUDIT  Follow up at 3 months: 65.9%. | At follow up, the CG: consumed an average of 20.08 drinks per week; BI consumed an average of only 17.19 drinks per week, representing a difference of 2.89 drinks per week. Among the CG: 69.23% reported heavy episodic drinking compared with 58.89% of the BI, 10.34 percentage points less. The average AUDIT score for the control group was 14.04 at follow up, while the average for the intervention group was 11.59. | Grade: 3/5 |

| Authors | Target Group | Design and Methods | Intervention Details | Outcome Measures:  Tools/Follow up | Main Results | Comments/Grading |
| --- | --- | --- | --- | --- | --- | --- |
| Longabaugh R, Woolard RE, Nirenberg TD, et al. *J Stud Alcohol.* 2001;62(6):806-810. USA | Level 1 TC ED, 18+, both genders, injured patients met inclusion criteria if they were assessed as hazardous or harmful drinkers by one of three criteria: AUDIT 8+, positive for alcohol (BAC ≥0.003 mg/dL) during their ED visit, or they reported having ingested alcohol in the 6 hours prior to their injury.  (N = 539) | Administered by interventionists: AUDIT (administered during screening), the DrInC and IBC.  Prior to initiating the intervention, the patient repeated the breath analyzer test when necessary to determine that his or her BAC was ≤0.10 mg/dL.  Patients were randomly assigned to SC(188); BI(182); or BIB (169): scheduled for a return visit to the hospital 7 to 10 days after the initial session. Each participant received $15.00. | Interventionist; SC; BI: @40-60 min. or BIB: scheduled for a return visit to the hospital 7 to 10 days after the initial session. | AUDIT, DrInC and IBC.  Only 95/169 (56%) returned for the booster session.  Follow up:  83% at 12 month follow up. | 78% were men and the average age was 27 years. The ethnic composition: 72% white, 14% Hispanic, 10% black, <1% Asian, <1% Native American, and 3% other. 77% single, and 72% were employed. AUDIT score of 12.8, DrInC lifetime negative consequences score of 15.6 and had averaged 1.6 self-reported alcohol-related injuries in the prior year. Patients receiving BIB, but not BI patients, reduced alcohol-related negative consequences and alcohol-related injuries more than did those in the SC group. All three groups reduced their HDD. Patients with histories of hazardous drinking responded to BIB, whether or not they had consumed alcohol prior to their injury. | Grade 2/5 |

| Authors | Target Group | Design and Methods | Intervention Details | Outcome Measures:  Tools/Follow up | Main Results | Comments/Grading |
| --- | --- | --- | --- | --- | --- | --- |
| Maio RF, Shope JT, Blow FC, et al. *Ann Emerg Med.* 2005;45(4):420-429. USA | Injured patients treated in Level I TC ED, 14‐18 years, both genders; identified by a research assistant, method unclear  (N =655) | BI (329), CG (326); adolescents identified by scanning the electronic ED log and checking with ED staff, then asked to participate, consent obtain from parents or guardian. | BI: After a laptop‐based baseline survey, adolescents used an interactive computer program, selected a cartoon character with whom they attended a virtual house party. Characters gave feedback and information depending on players’ actions.  "The essential content included increasing knowledge about alcohol (...), increasing refusal skills, and decreasing intentions to misuse alcohol."  Adolescents received a message based on their responses to alcohol use items and a keychain with a picture of their party pal.  CG: Laptop‐based baseline survey only | Amidx, Alcohol Frequency/Quantity Index  Follow up: 3 months (89%), 12 month (89%), telephone contacts, same as baseline. | No significant effects of BI. Alcohol‐related negative Consequences (Amidx) decreased at 3 months but returned to baseline at 12 months for BI and CG. Binge drinking decreased at 3 months and returned to baseline at 12 months (BI), did not change for CG.  Beneficial effect of BI among adolescents with previous drinking and driving (small group). |  |
| Mello MJ, Longabaugh R, Baird J, et al. *Ann Emerg Med.* 2008;51(6):755-764. USA | Injured patients treated in Level 1 TC ED and 2 community hospital EDs, DIAL project, 18+ years, both genders; screened positive for at-risk drinking according to the NIAAA quantity frequency guidelines (for men: 15 drinks/week or 5+ drinks/occasion; for women: 8+ drinks/week or 4+drinks/occasion). (N =285) | BI (140), CG (145); patients screened positive for at-risk drinking (NIAAA criteria) were asked to participate.  Assessment, enrollment, and randomization were done by telephone call in the day after discharge. | BI: Certified MI counselors delivered 1 30‐ minute MI session by telephone 5 days after discharge, and 2 weeks later a 15‐minute  BI booster session. Counselors were master´s or doctoral‐level staff.  CG: no study intervention | AUDIT, Impaired Driving Scale, other high‐risk driving behaviors (telephone contacts)  Follow Up: 3 months (96%) same as baseline (telephone contacts). | At 3‐month follow‐up no significant differences between BI and CG for AUDIT scores, both groups showed a decrease of 30%. The participants with AUDIT scores >15 in BI group had a lower 3‐month impaired driving score. | Grade 3/5 |

| Authors | Target Group | Design and Methods | Intervention Details | Outcome Measures:  Tools/Follow up | Main Results | Comments/Grading |
| --- | --- | --- | --- | --- | --- | --- |
| Mello MJ, Baird J, Lee C, Strezsak V, French MT, Longabaugh R. A Randomized Controlled Trial of a Telephone Intervention for Alcohol Misuse with Injured Emergency Department Patients. *Annals of emergency medicine*. 2016;67(2):263-275. USA | Two EDs: level I TC and an academic community ED;  age ≥ 18 years old, ED visit was for an injury, English speaking and medically stable, not being admitted to the hospital and patient was not incarcerated or intoxicated.  (N=730) | RA (Research Assistants) identified the patients and the patients completed an Alcohol, Smoking and Substance Involvement Screening Test (ASSIST); ASSIST score of 11 or greater (moderate or high-risk alcohol use) randomized to telephone intervention on either alcohol use or home fire and burn safety.  Participants received $20 for completion of initial assessments in the ED, $20 for each intervention call, and $40 for completion of subsequent outcome assessments at 4, 8 and 12 months. | ED patients were randomized: 78% received by telephone three-session telephone brief motivational intervention (TBMI) delivered over 6 weeks by a total of six interventionists trained in BMI; the control group received 3 calls by the RAs. After each intervention, a second RA called all participants in both groups to complete a post-intervention call assessment of the participants’ experience of the intervention using the Participant Rating Form. | The follow-up interview: Alcohol consumption questions included typical quantity (drinks per occasion) and frequency (days per week) of alcohol use. DrInc SIP, alcohol-related injuries and arrests for drinking and driving.  Follow up: At 12 months 72% completed (70% TBMI; 75% HS). | No difference between TBMI and CG. Participants in both groups improved, with decreased self-reported alcohol consumption and impaired driving at each of the follow-up assessment points following the intervention, relative to prior to the intervention. The greatest improvement in reduction in consumption was reported at 4 months post intervention, and maintained through to the 8 month follow up point. While there was some increase in consumption from 8 to 12 months, at 12 months participants remained at lower levels of alcohol consumption and impaired driving in comparison with baseline rates. | 3/5 |
| Monti PM, Colby SM, Barnett NP, et al. *J Consult Clin Psychol.* 1999;67(6):989-994. USA | ED following an alcohol-related event or BAC+, patients 18 to 19 years.  (N = 94) | ADI, ADQ; Patients assigned to either MI (52): done by interventionists or SC(42): received a handout on avoiding drinking and driving and a list of local treatment agencies. Patients received $20 at baseline; $10 at 3 months; and $15 at 6 months follow up. | BI: Trained clinicians (Bachelor’s or master´s level students) delivered 1 MI session 35-40 min. (Several MI sessions were audiotaped.)  SC: 5 min. | Follow up: At 3 months 93% by telephone;  At 6 months 89 % in person | Follow-up assessments showed that patients who received the MI had a significantly lower incidence of drinking and driving, traffic violations, alcohol-related injuries, and alcohol-related problems than patients who received SC. Both conditions showed reduced alcohol consumption. The harm-reduction focus of the MI was evident in that MI reduced negative outcomes related to drinking, beyond what was produced by the precipitating event plus SC alone. | Grade 2/5 |

| Authors | Target Group | Design and Methods | Intervention Details | Outcome Measures:  Tools/Follow up | Main Results | Comments/Grading |
| --- | --- | --- | --- | --- | --- | --- |
| Monti PM, Barnett NP, Colby EM, et al.  *Addiction.* 2007;102(8):1234-1243. USA | Injured patients treated in Level 1 TC ED, 18‐24 years, both genders; BAC > 0.01% or reported drinking 6 hours prior to the event or AUDIT score 8+.  (N =198) | BI (98), CG (100); patients who screened positive were asked to participate, and counselors administered baseline assessment using a laptop computer (30‐45 minutes) | BI: A computer‐generated personalized feedback report was delivered and discussed, using MI techniques (30‐45 min.). Patients received copies of feedback report and worksheets. At 1 month, a telephone booster session (20 min.) was conducted, including 30‐day TFLB. For the 3‐month booster (25‐30 min.) patients completed a short assessment battery of alcohol consumption. A new feedback sheet was generated and discussed, and send to the patient. CG: Patients received the same handouts as in MI; the contact with the counselor lasted 1‐3 min. At 1 month, the telephone contact consisted only of the 30‐day‐TLFB. At 3 months, patients completed the same assessment battery as MI patients (10‐15 min) and were send a new feedback sheet. | TLFB 30 days prior to ED visit, RAPI, alcohol related questions (# of days drinking, HED, average drinks per week), AIC, counseling or treatment for alcohol, adverse driving events (registry data) 1 year pre ED admission.  Follow Up: 6 months (83%), 12 months (81%); same as baseline, adverse driving events (registry data) 1 year post ED admission | Both groups had significantly reduced the # of drinking days, the # of heavy drinking days and the average # of drinks per week. A treatment x time interaction was found on all three consumption measures for 6‐ and 12‐month follow‐up.  At 12 months (reduction rates) # day drinking: 45% (BI), 11% (CG); # heavy drinking: 51% (BI), 12% (CG); # drinks per week: 53% (BI), 18% (CG). RAPI scores: no treatment effect  Alcohol‐related injuries: no treatment effect, time effect. | Boosters may have strengthened the effect. Reduced alcohol-related negative consequences could be a reaction to the trauma, to the overall treatment in ED, or to the assessment.  Grade 3/5 |
| Roudsari B, Caetano R, Frankowski R, et al. *Ann Emerg Med.* 2009;54(2):285-293. USA | Injured patients treated in Level 1TC ED, 18+ years, both genders;  4 criteria: positive BAC or self-reported drinking within 6 hours before injury, drinking pattern beyond NIAAA standards, 1+ positive item on CAGE. (N = 1,493) | BI (737), CG (756) patients screened positive on one or more criteria were asked to participate. | BI: Trained clinicians (master´s level students) delivered 1 MI session. (Several MI sessions were audiotaped.)  CG: same assessment as BI (30‐40 minutes) | Follow Up: 6 months (69%), 12 months (58%) same as baseline (telephone contacts) | No statistically significant association between the intervention and injury outcomes at 6 and 12 months.  No significant association between brief intervention and injury recidivism.  Ethnicity did not have a modification effect. |  |

| Authors | Target Group | Design and Methods | Intervention Details | Outcome Measures:  Tools/Follow up | Main Results | Comments/Grading |
| --- | --- | --- | --- | --- | --- | --- |
| Segatto ML, Andreoni S, de Souza e Silva R, et al. *Rev Bras Psiquiatr.* 2011;33(3):225-233. Brazil | Injured patients treated in ED, 16‐25 years old, both genders; treated for alcohol-related events and admitted to ED up to 6 hours after last alcohol use  (N= 175) | BI (87), CG (88); Screened positive (alcohol related events and admitted to ED up to 6 hours after alcohol use) were asked to participate. | BI: Trained senior psychologist delivered 1 45‐minute MI session. Patients received an educational brochure (ED) of 3 pages on the risks of alcohol consumption and possible tips to reduce alcohol and avoid negative consequences.  CG‐ED: Patients received an educational brochure (ED) | SCID‐1/P, DSM‐IV (use, abuse and dependency), ACQ, RAPI, ACRQ, APRA, RTCQ  Follow Up: 3 months (85%); same as baseline, either face‐to‐face in ED, by telephone call or home visit. | At 3 months no significant differences between BI and CG concerning all outcome measures.  Overall, statistically significant decrease in days of alcohol use, days with moderate use, days with heavy use, and negative consequences. | Experiencing an alcohol-related ED visit itself was the trigger to modify short‐term alcohol consumption.  Grade 3/5 |
| Spirito A, Monti PM, Barnett NP, et al. *J Pediatr*. 2004;145:396-402. USA | Injured patients treated in ED 13‐17 years, both genders, evidence of alcohol in blood, breath or saliva or reported drinking alcohol in the 6 hours before the injury  (N=152) | BI (78), CG (74); screen positive were asked to participate, written informed consent from parents. | BI: 12 MI trained bachelor´s and master´s  level interventionists delivered 1 35‐ to 45‐minutes MI session.  CG: short contact (5 minutes) with physicians included brief advice to stop drinking and avoid drinking and driving. | ADQ, ADI, Young Adult Drinking and Driving Questionnaire, AIC, and MAST (parents).  Follow Up: 3 months by telephone (93,4%), 6 months face‐to‐face (89,5%),  12 months face‐to‐face (89,5%): ADQ, Young Adult Drinking and Driving Questionnaire, AIC, Adolescent Health Behavior Questionnaire. | "The intervention group main effects and the group by time interactions were non significant for all variables."  For adolescents with high ADI‐Scores only (scoring above the clinical cutoff): Significant differences between groups at follow‐up. Those receiving BMI rather than short contact (CG) had fewer drinking days per month and fewer high-volume drinking days per month. | The alcohol‐related event and having treated in the ED may have been sufficient for adolescents with low levels of alcohol involvement. Intensity of screening may have an effect on adolescents in CG  Involvement of parents should be taken into account.  Grade 3/5 |

| Authors | Target Group | Design and Methods | Intervention Details | Outcome Measures:  Tools/Follow up | Main Results | Comments/Grading |
| --- | --- | --- | --- | --- | --- | --- |
| Spirito A, Sindelar‐Manning H, Colby SM, et al. *Arch Pediatr Adolesc Med*.2011;165(3):269-274. USA | Injured patients treated in Level 1 TC ED, 13‐17 years, both genders; evidence of alcohol in blood, breath, or saliva or reported drinking alcohol in the 6 hours before the injury.  (N =125) | BI‐IMI (63), BI‐IMI+FCU (62); patients screened positive were eligible for the study and received an individual motivational interview (IMI) before randomization. (Patients received $20.)  MI session, focused on personal responsibility, personalized assessment feedback, anticipating barriers, developed discrepancy between current drinking and longer term goals, support self efficacy.  The Family Check‐Up (FCU) is an assessment and feedback intervention. | BI-IMI: trained interventionists delivered 1 45 min. BI‐IMI‐FCU: patients received MI intervention. Families returned for 1 1-hour family assessment task (FAsTask). Parents and adolescents discussed family beliefs regarding substances, and other topics. Patients received feedback in a second 1‐hr session. (Parents received $50.) Patients in both conditions were given f/up information regarding substance use treatment services and received 5 monthly booster brochures on parenting before the 6‐month follow‐up. | ADQ  Follow up: 3 months (78%), 6 months (74%), 12 months (66%); same as baseline, 3 months by telephone, 6 and 12 months in person by research assistant. | At 3, 6 and 12 months no statistically significant differences (except for high volume drinking days at 3 months, BI‐IMI‐FCU group fewer days) between the intervention conditions.  Significant results for the sample as a whole (baseline compared with 3,6,and 12months: a) # of drinking days per month, b) quantity per drinking occasion, c) high‐volume drinking days (84% at baseline, 24% at 3 months, 35% at 6 months, 53% at 12 months).  Any drinking in the previous month 100% at baseline, 39% at 3 months, 55% at 6 months, 68% at 12 months. | "Return visits to complete  FCU sessions  proved problematic for approximately  20% of the families in this study."  Grade 2/5 |

| Authors | Target Group | Design and Methods | Intervention Details | Outcome Measures:  Tools/Follow up | Main Results | Comments/Grading |
| --- | --- | --- | --- | --- | --- | --- |
| Stein LA, Minugh PA, Longabaugh R, et al. *Psychol Addict Behav.* 2009;23(2):185–195**.** USA | Level 1 TC ED. 18 +, 78% were men, 72% white, 14% Hispanic, 10% African American, <1% Asian, <1% Native American, and 3% other ethnicities or races; the average age was 27 years (SD = 9) with an injury that did not result in admission to the hospital, and received a hazardous drinking assessment:  BAC ≥0.003 mg/dL or reported drinking 6 hours prior to the event or AUDIT score 8+.  (N = 539) | The interventionist approached injured patients and asked permission to screen them and to explain the study briefly. The interventionist administered the AUDIT to patients who agreed.  Each participant received $15 for completing the baseline instruments. Prior to intervention assignment, the patient was retested to ensure that BAC was ≤0.1 mg/dL. Assessment: AUDIT, the DrInC, and a decision ladder.  Randomized into three conditions: (a) standard care plus assessment (SC), (b) SC plus BMI (BI), and (c) BI plus a booster session (BIB). | SC: received the same customary care.  BI: occurred after the baseline assessment was completed and prior to the patient being discharged from the hospital. The session lasted 40–60 min. The intervention was based on motivational enhancement treatment implemented in Project MATCH.  BIB: return appointment scheduled within 7-10 days of the initial session. | Patients returned to the hospital for follow-up interview visits. Those unwilling or unable to do so were offered the option of completing the follow-up questionnaire at an off-site location or by a combination of telephone and mail. Patients were compensated $25 for each completed interview.  Follow up: at 1 year, 417 (77.4%) of the those that completed the 3 month followed up | At 12-month follow up BIB patients had significantly reduced alcohol consequences 17.2 (SD = 19.5; range= 0–135), more than had SC patients, which is comparable to normative 12-month scores for the DrInC.  Patients receiving BI or BIB maintained higher readiness scores 3 months after treatment than did patients receiving SC. However, readiness mediated treatment effects only for those highly motivated to change prior to the intervention but not for those with low pre-intervention motivation. | Grade 3/5 |
| Suffoletto B, Callaway B, Kristan J, et al. *Alcohol Clin Exp Res.* 2012;36(3):552-560. USA | 3 EDs: 2 Level 1 TC ED; 1 ED; injured patients treated in ED, "PART" Pittsburgh Alcohol Reduction Through Text‐Messaging, 18‐24 years, both genders; AUDIT‐C score 4+ (men), 3+women  (N = 45) | BI (15) CG‐A (Assessment, 15), CG (15); patients identified as hazardous drinkers (AUDIT‐C) were asked to participate. | A 16‐page booklet published by the NIAAA, entitled "Rethinking Drinking".  BI: assessment of the amount of alcohol intake. Second text message with feedback  CG‐A: assessment of the amount of alcohol intake.  CG: Each week of 12 weeks patients were reminded of the final survey. | TLFB, RAPI, willingness and confidence to reduce alcohol use (VAS), Sociodemographic data and drinking variables.Participants self-administered assessment using a tablet computer.  Follow Up: 3 months (87%); same as baseline + questions re: the use of the booklet and comfort of text messages, web‐based questionnaire. | At 3 months significant differences were observed between BI group and CG‐A only, considering the fact that CG‐A increased their drinking over the course of the study.  Statistically significant result at 3  months: a) Change in # HDDs in last month: BI (‐ 3.4), CG‐A (1.8), CG (‐ 1.1) b)# DPDD in last month: BI (3.1), CG‐A (5.2), CG (3.8) c) Changes in # DPDD in last month: BI (‐ 2.1), CG‐A (1.1), CG (‐ 0.6). | Grade 3/5 |

| Authors | Target Group | Design and Methods | Intervention Details | Outcome Measures:  Tools/Follow up | Main Results | Comments/Grading |
| --- | --- | --- | --- | --- | --- | --- |
| Trinks A, FestinK, Bendtsen P, et al. *Int Emerg Nurs*. 2010;18(3):138-146. Sweden | Injured patients treated in ED, 18‐69 years, both genders; risky drinkers, weekly consumption of 10+ drinks (women) or 15+ drinks (men) and/or HED (4+ drinks, women, 5+ drinks, men; on one occasion) once a month or often.  (N = 93) | BI‐L (long feedback, NR), BI‐S (short feedback, NR). All eligible (ie, not too ill) were requested to answer alcohol‐related questions on a touchscreen computer. 560 patients were categorized as risky drinkers and asked if they would be willing to respond to a follow‐up postal questionnaire. 560 patients categorized as risky drinkers, 415 unwilling to be followed up, 52 non‐responders, 93 responders. | BI‐L: After completed the computer program they received a one‐page printout with tailored advice and information concerning their drinking patterns and motivation to change behavior. A graphic illustration showed the risk levels regarding their alcohol use.  BI‐S: Patients received only the graphic illustration based on their alcohol use. | Three outcome measures were used: (1) Participation: patients who initialized and completed the computerized test, willingness to follow up 6 months later, and follow-up questionnaire. (2) Representativeness was examined by comparing age, sex, socio-demographic and alcohol data for patients who initialized but did not complete the computerized test with those who completed. (3) Effectiveness was measured by comparing the long and short feedback conditions in terms of absolute and relative changes, from baseline to follow up, in weekly alcohol consumption (in grams) and number of HED occasions per month, and the proportion of patients who changed from risk drinking to non-risk drinking levels. | At 6 months no statistically significant differences between BI‐L and BI‐S groups.  Weekly alcohol intake decreased by 34% (BI‐L) and by 26% (BI‐S), frequency of HED decreased by 40% (BI‐L) and by 33% (BI‐S). 56% (BI‐L) and 39% (BI‐S) of the risky drinkers at baseline became non risky drinkers at 6‐month follow up. | Grade 2/5 |

| Authors | Target Group | Design and Methods | Intervention Details | Outcome Measures:  Tools/Follow up | Main Results | Comments/Grading |
| --- | --- | --- | --- | --- | --- | --- |
| Walton MA, Goldstein AL, Chermack ST, et al. *J Stud Alcohol Drugs.* 2008;69(4):550–560. USA | Injured patients age 19+ presenting to Level 1 TC ED, completed a computer-based survey of health issues. Met at-risk drinking criteria based on either average consumption in the past 3 months (15 or more drinks/week for men age <65; 12 or more drinks/week for women age <65 and men age ≥65; and 9 or more drinks/week for women age ≥65) or heavy drinking (5 or more drinks/occasion on at least four occasions in the last month [eg, weekly heavy drinking] for men age <65, and 4 or more drinks/occasion on at least four occasions in the past month for younger women [age <age 65] and for men and women age ≥65. Alcohol within 6 hours before their injury. Readiness to change was assessed using two questions: “Do you plan to cut down on your drinking in the next 30 days?” and “Do you plan to cut down on your drinking in the next 6 months?”  (N=575) | Four intervention conditions:   - advice + tailored booklet, - advice + generic booklet, - no advice + tailored booklet, - or no advice + generic booklet. | All therapists had master’s degrees in social work or related field.  During the advice session, the tailored or generic booklet was reviewed with the participant prior to discharge from the ED. No advice conditions were given either a tailored or generic booklet to review. | The follow-up interview included identical questions to that of the baseline assessment. Alcohol consumption questions included typical quantity (drinks per occasion) and frequency (days per week) of alcohol use. DrInc SIP, alcohol-related injuries and arrests for drinking and driving.  Follow up:  3 and 12 months (85%) | Overall, participants who reported higher levels of self-efficacy had lower weekly consumption and consequences whereas those with higher readiness to change had greater weekly consumption and consequences.  Individuals who attributed their injury to alcohol and received advice had significantly lower levels of average weekly alcohol consumption and less frequent heavy drinking from baseline to 12-month follow up compared to those who attributed their injury to alcohol but did not receive advice. |  |

| Authors | Target Group | Design and Methods | Intervention Details | Outcome Measures:  Tools/Follow up | Main Results | Comments/Grading |
| --- | --- | --- | --- | --- | --- | --- |
| Wang TC, Kyriacou DN, Wolf MS. *J Emerg Med.* 2010;39(5):561-568. USA | Injured patients treated in ED, 18‐75 years, both genders, identified and selected by the principal investigator  (N= 252) | BI (125), CG (127); screened positive by the principal investigator were asked to participate. | BI: The brochure titled "Alcohol, How Much is Too Much?" was given to the patients. The brochure was developed by the American College of Emergency Physicians (ACEP) as part of a resource kit for use in the ED.  CG: no brochure handed over to the patients. | BI group and CG participated in a standard interview by the principal investigator, using standardized, scripted, closed ended questions. AUDIT‐S and RTC‐Q (short form) were embedded and knowledge of safe alcohol use was tested.  Follow Up: 1 month (75%); telephone contact by principal investigator. | At 1 month no significant decreases in HED or knowledge of safe drinking could be found. No significant differences between BI group and CG were observed. A significant increase in readiness‐to-change was found in the BI group, but not in the CG (in excessive alcohol users, AUDIT 2+ for men and 1+ for women). | Grade 2/5 |
| Woolard R, Baird J, Longabaugh R, et al. *Addict Behav*. 2013;38(3):1732–1739. USA | Level 1 TC, research assistants screened 18+ years patients using the Wellness questionnaire.  (N=515) | BIB (239), CG (266), after the initial screening alcohol and marijuana consumption was evaluated with AUDIT and alcohol, and marijuana and drug use index (AMD); Noteworthy Index of Problems (NIP) was developed and adapted from the DrInC; and IBC.  Treatment consisted of two sessions of BI, by trained interventionists.  At 3 and 12 months, both groups had an assessment of alcohol and marijuana use and negative consequences of use. | BIB: Treatment consisted of two sessions of BI, one in the ED and a second BI within 2 weeks of presentation conducted by trained interventionists. The first BI session lasted between 20 and 60 min, and the second 15 to 40 min. In addition, participants were offered information on local treatment resources for substance misuse  CG: Participants were offered information on local treatment resources for substance misuse. | 51% of participants in the BI group returned for the booster session.  Follow up:  85% at 6 months  83% at 12 months | Measures of binge drinking and conjoint marijuana and alcohol use significantly decreased for BI as compared to the CG. At 12 months binge alcohol use days per month in BI was 0.72 (95% CI=0.36–1.12) compared to CG 1.77 (95%CI=1.19–1.57) Conjoint use days in the treatment group 1.25 (95%CI=0.81–1.54) compared CG 2.16 (95% CI=1.56–2.86). No differences in negative consequences or injuries were seen between BI and CG. | Grade3/5 |

ACQ=Alcohol Consumption Questionnaire; ACRQ=Alcohol Consumption Risk Questionnaire; ADI=Adolescent Drinking Index; ADQ =Adolescent Drinking Questionnaire; AIC=Adolescent Injury Checklist; APRA=Alcohol Perception of Risk Assessment; BAC=Blood Alcohol Concentration; BI= Brief Intervention; BIB=Brief Intervention plus a Booster Session; BI‐IMI=Individual Motivational Interview; CG= Control Group; DSM‐IV= 4th revised edition of the Diagnostic and Statistical Manual of Mental Disorders; DPDD=Drinks per Drinking Day; DrInC= Drinkers Inventory of Consequences; FCU =Family Check-Up; HDD=heavy drinking days; IBC=Injury Behavior Checklist; MPH=Masters in Public Health; RAPI= Rutgers Alcohol Problems Index; RTCQ= Readiness to Change Questionnaire; SCID‐1/P=Semi-structured Clinical Interviewing; SMAST=Short Michigan Alcohol Screening Test; TAU+= Treatment As Usual with Assessment; TC=Trauma Center; TLFB= Timeline Follow-back.
