# Supplementary material for: Effectiveness of SBIRT for Alcohol Use Disorders in the Emergency Department: A Systematic Review
Source: West J Emerg Med. 2017 Sep 21;18(6):1143–52. doi: 10.5811/westjem.2017.7.34373 (PMC5654886; doi:10.5811/westjem.2017.7.34373)
Supplement: Supplementary file 2 [file wjem-18-1143-s002.docx]

Appendix B. Screening Tools for Alcohol Disorders

| Screening tool & Studies  Using the tool |  | Description | Sensitivity and Specificity |
| --- | --- | --- | --- |
| **AUDIT**  A score of 8 or higher,  ^28, 31-33, 38, 39, 42, 50, 53, 54^  Two studies stratified the patients into three categories: 0 to 6 (low risk), 7 to 18 (at-risk/moderate risk), and 19 to 40 (high risk). ^23, 24^ | AUDIT (Alcohol Use Disorders Identification Test) was developed by the World Health Organization as a simple tool to screen for excessive drinking and to assist in brief assessment in primary care settings. AUDIT is a tool to identify persons with hazardous and harmful patterns of alcohol consumption, as well as alcohol dependence. Items include alcohol consumption quantity and frequency measures, heavy drinking, dependence symptoms, tolerance, and alcohol-related negative consequences. ^59,60^ | The l0 items have shown high sensitivity and specificity (with a weighted score of 8 or more) in an extensive six-nation validation trial.^58^ It is particularly designed for health care practitioners and a range of health settings, but with suitable instructions it can be self-administered or used by non-health professionals. It takes only about 5 minutes to complete, has been tested internationally in primary care settings, and has high levels of validity and reliability.^59^ | The AUDIT has great internal consistency (alpha is .80), and it reliably distinguishes between patients with hazardous and harmful drinking histories and those without such histories.^60^ The AUDIT shows an optimal balance of sensitivity and specificity for detecting all forms of alcohol misuse when cutoff points of 4 or more (sensitivity 84% to 85%; specificity 77% to 84%) or 5 or more (sensitivity 70% to 92%; specificity 73% to 94%) are used; use of higher cutoff points increases specificity to an extent but reduces sensitivity.^61^ |
| **AUDIT-Consumption (AUDIT-C)**  Used by ^42^ |  | The AUDIT-C questionnaire is a 3-item self-report measure that assesses the quantity and frequency of average alcohol consumption and the frequency of binge drinking episodes in the prior 12 months.^62^ | The sensitivity and specificity of AUDIT-C are best balanced at cutoff points of 4 or more (74% to 76% and 80% to 83%, respectively) and 3 or more (74% to 88% and 64% to 83%, respectively). ^62^ |
| **CAGE**  Six studies  ^21-24, 35, 39^ used the CAGE questionnaire to screen injured patients for alcohol consumption;  AUDIT was used with another alcohol screening tool such as CAGE, ^23, 39^ | The CAGE screening tool focuses on behaviors consistent with alcohol dependence | CAGE is a mnemonic designed for rapid verbal screening in a clinical setting and is derived from the following four items. (1) Have you ever felt you should cut down on your drinking? (2) Have people annoyed you by criticizing your drinking? (3) Have you ever felt bad or guilty about your drinking? (4) Have you ever had a drink first thing in the morning to steady your nerves or get rid of a hangover (eye opener)? | Sensitivity and specificity for this assay have been found to be 72% and 79%, respectively (on the basis of positive responses to two or more of the items) compared with psychiatric evaluation when applied to a Department of Veterans Affairs psychiatric inpatient population. ^63, 64^ It is not sensitive for detecting heavy drinking and does not distinguish between active and past problem drinking. ^65, 66^ |
| **PAT**  Two studies, one in the United Kingdom and one in Australia, employed the PAT. ^29, 34^ | The Paddington Alcohol Test (PAT) is a clinical and therapeutic tool for selective Early Identification and Brief Advice (EIBA) of both hazardous and dependent drinking. | Paddington alcohol test (PAT) has been refined overtime to include the following questions: if patient drinks, what is the most the patient drinks in one day, if it is more than double the safe limit for one day (8 units for a male and 6 units for a female), then ask how often they drink that much (to differentiate hazardous [binge], harmful versus dependent drinking), and the last question is whether in the patients' view their visit to the emergency department as being alcohol related. ^67,68^ The PAT is targeted at those patients who present with conditions associated with alcohol misuse and it has been condensed into 10 conditions: fall, collapse, head injury, assault, accident (burn), request detox, non-specific GI symptoms, psychiatric, cardiac (chest pain), repeat visits ^69^ The PAT was specifically developed for use in a busy ED, to make best use of the “teachable moment.” ^70,71^ |  |
| **MAST**  Used by ^48^ | The original MAST is widely accepted in screening for alcoholism in both clinical and nonclinical settings, and its validity, reliability and internal consistency are well established.^72^ | The Michigan Alcoholism Screening Test (MAST) has 25 questions that address the consequences of alcohol use, and focus both on current and past problems. ^72, 73^ |  |
| **Brief-Mast** |  | The brief-MAST (Michigan Alcoholism Screening Test) is a 10-item subset of the original 25-item MAST. | The brief-MAST when scored according to the MAST with applied weights of 1, 2, and 5 correlates well (range, .95 to .99) with MAST scores and discriminates equally well between alcoholic and nonalcoholic psychiatric patients when a cutoff score of 6 or more is used. ^74^ |
| **TWEAK** | The TWEAK screening tool was developed to identify hazardous drinking among women. | The TWEAK tool is a mnemonic for questions having to do with tolerance- (patient can hold six or more drinks), friends or relatives worried about the patient's drinking, taking a drink first thing in the morning (eye-opener), blackouts (amnesia), and a self-perceived need to cut down on drinking. ^75^ Two of the items, eye opener and cutting down, were taken from the CAGE; two others, worriedness and amnesia, were taken from the MAST. | Although the TWEAK assay was initially designed to identify “at-risk” pregnant drinkers, it has been found to have high sensitivity and specificity in both primary care and general populations, ranging from 83% to 100% and 68% to 96%, respectively, using a cutoff point of 3 when a weight of 2 is applied to tolerance and worry and a weight of 1 is applied to the remaining three items. ^75^ |
| **NIAAA**  AUDIT was used with other alcohol screening tool such as NIAAA ^28 66^ | The tool can be used to detect heavy drinking (no abuse or dependence) or to assess for alcohol abuse disorders (abuse or dependence). | Patients are at increased risk for alcohol-related problems, men who drink more than 4 standard drinks in a day (or more than 14 per week) and women who drink more than 3 in a day (or more than 7 per week). (One standard drink is equivalent to 12 ounces of beer, 5 ounces of wine, or 1.5 ounces of 80-proof spirits). ^76^ | Single-question screening has a reported sensitivity of 82% to 87% and specificity of 61% to 79%. ^76^ |
