# Supplementary material for: Effectiveness of SBIRT for Alcohol Use Disorders in the Emergency Department: A Systematic Review
Source: West J Emerg Med. 2017 Sep 21;18(6):1143–52. doi: 10.5811/westjem.2017.7.34373 (PMC5654886; doi:10.5811/westjem.2017.7.34373)
Supplement: Supplementary file 3 [file wjem-18-1143-s003.docx]

Appendix C. Adolescent Screening Tools for Alcohol Disorders and Consequences of Drinking

| Screening Tool | Description |
| --- | --- |
| **Adolescent Drinking Questionnaire (ADQ)**  Three studies involving adolescents used Adolescent Drinking Questionnaire (ADQ), and Adolescent Drinking Index (ADI) instruments to evaluate alcohol consumption ^44, 48, 51^ | The ADQ consists of 4 items that assess recent drinking frequency (days per month), quantity (drinks per occasion), frequency of high-volume drinking (>5 drinks per occasion), and frequency of intoxication (feeling ‘‘drunk’’ or ‘‘very, very high’’) over the prior 3 months. Each item is scored on an 8-point scale. ^77^ |
| **The Adolescent Drinking Inventory (ADI)**  Used by ^44^ | ADI is a 24-item measure of the severity of alcohol involvement. Items are scored on either a 3-point scale from 0 (not like me at all) to 2 (like me a lot), or on a 4-point frequency scale from 0 (never) to 3 (>4 times). ^78^ |
| **The Young Adult Drinking and Driving Questionnaire**  Used by ^41^ | The Young Adult Drinking and Driving Questionnaire consists of 5 items measuring the frequency of driving after doing any drinking at all, driving within an hour of consuming 1 to 2 drinks, driving within an hour of consuming >3 drinks, drinking while driving, and riding with a driver who had been drinking or using drugs.^79^ |
| **World Health Organization's Alcohol, Smoking and Substance Involvement Screening Test (ASSIST)**  Used by ^43^ | The ASSIST is an 8-item questionnaire given by a healthcare worker to a patient using paper and pencil, and it can be completed in about 5-10 minutes. The ASSIST was designed to screen for the use of alcohol, tobacco, and other substances of abuse.  The ASSIST determines a risk score of ‘lower‘, ‘moderate’ or ‘high’ risk category for each substance. ^80,81^ |
| **Alcohol Misuse Index (Amidx)** | The Amidx is a validated, 10-item measure quantifying the negative consequences of drinking alcohol. It includes questions on how many times in the previous 3 months they got drunk, they drank more than planned, they got sick, they got into trouble with friends, a friend of same sex complained, a friend of opposite sex complained, a date complained, they got into trouble with their parents, they got into trouble at school or work, or they got into trouble with the police. The value of the Amidx represents the number of episodes of alcohol misuse within the past 3 months. The possible range of values is 0 to 60; higher scores mean more misuse. ^82, 83^ |
| **Family Check-Up (FCU)** | The Family Check-Up (FCU) is a brief, family-centered intervention focused on family-management practices. ^84^ |
| **Short Michigan Alcoholism Screening Test (SMAST)**  Used in these studies ^44, 48, 51^ | The Short Michigan Alcoholism Screening Test (SMAST) ^85^ is a 13-item report completed by the parents to detect alcohol problems. A score of 5 indicates suspected alcoholism. |
